# Prevention and Management of Perioperative Acute Kidney Injury: A Narrative Review

**DOI:** 10.3390/diseases13090295

**Published:** 2025-09-05

**Authors:** Mary O’Dell Duplechin, Garrett T. Folds, Drake P. Duplechin, Shahab Ahmadzadeh, Sarah H. Myers, Sahar Shekoohi, Alan D. Kaye

**Affiliations:** 1 School of Medicine, Louisiana State University Health Sciences Center at Shreveport, Shreveport, LA 71103, USA; mmo002@lsuhs.edu (M.O.D.); gtf001@lsuhs.edu (G.T.F.); dpd001@lsuhs.edu (D.P.D.); 2 Department of Anesthesiology, Louisiana State University Health Sciences Center at Shreveport, Shreveport, LA 71103, USA

**Keywords:** perioperative acute kidney injury, prevention, management, surgical complications, nephrotoxic agents, risk stratification

## Abstract

Acute kidney injury is a common complication in the perioperative setting, especially among patients undergoing high-risk surgeries such as cardiac, abdominal, or orthopedic procedures. Characterized by a sudden decline in renal function, perioperative acute kidney injury is typically diagnosed based on rising serum creatinine or reduced urine output. Its incidence varies depending on the surgical type and patient risk factors, but even mild cases are linked to significant consequences, including prolonged hospital stays, enhanced healthcare costs, and higher mortality rates. Despite advances in surgical and anesthetic care, acute kidney injury remains a major cause of morbidity. The development of acute kidney injury in the perioperative period often results from a complex interplay of hypoperfusion, ischemia–reperfusion injury, inflammation, and exposure to nephrotoxic agents. While some predictive models and biomarkers, such as neutrophil gelatinase-associated lipocalin (NGAL), have shown promise in identifying patients at risk, widespread adoption remains inconsistent, and standardized prevention protocols are lacking. This narrative review synthesizes current evidence on the pathophysiology, risk factors, and prevention strategies for perioperative acute kidney injury. It explores emerging tools for risk stratification and early diagnosis, including novel biomarkers and learning-based models. Additionally, it highlights pharmacologic and non-pharmacologic measures to reduce acute kidney injury incidence, such as balanced fluid management, renal-protective anesthetic strategies, and bundle-based care approaches. Emphasizing a multidisciplinary and personalized model of care, this review highlights the need for coordinated efforts between anesthesiologists, surgeons, and nephrologists to identify modifiable risks and improve outcomes. Reducing the incidence of perioperative acute kidney injury has the potential to enhance recovery, preserve long-term kidney function, and ultimately improve surgical safety.

## 1. Introduction

Acute kidney injury (AKI) is a significant and potentially life-threatening complication in the perioperative setting, defined by an abrupt decline in renal function. Clinically, acute kidney injury is diagnosed by an increase in serum creatinine levels or a decrease in urine output over a short period. This condition is particularly prevalent among patients undergoing high-risk surgical procedures, including cardiac surgery, major abdominal operations, and orthopedic interventions. The incidence of perioperative acute kidney injury varies considerably depending on the type of surgery performed and patient-specific risk factors. For instance, a comprehensive meta-analysis encompassing 19 studies reported that postoperative acute kidney injury occurs in approximately 13.4% of surgical cases [1]. Importantly, acute kidney injury in this context is strongly associated with adverse outcomes, including a 12.6-fold increased risk of short-term mortality, prolonged hospitalization, and a higher frequency of complications affecting organs beyond the kidneys [1].

Despite advances in surgical techniques, anesthesia, and perioperative monitoring, the occurrence of acute kidney injury remains alarmingly common and continues to worsen patient prognoses. Acute kidney injury complicates 5–7.5% of all hospital admissions and contributes to up to 20% of ICU cases, with 30–40% of in-hospital acute kidney injury events arising in surgical contexts [2]. Even mild forms of acute kidney injury are clinically significant, as they have been linked to longer hospital stays and increased healthcare costs. In addition to the perioperative period, the postoperative period also poses a high risk for acute kidney injury, primarily related to complications such as hypotension and sepsis [3].

Given the clinical and economic burden imposed by perioperative acute kidney injury, early recognition and prevention strategies are critically important. Approaches to prevention highlight the importance of minimizing exposure to nephrotoxic medications, hemodynamic monitoring to maintain adequate renal perfusion, and the utilization of emerging renal biomarkers, such as neutrophil gelatinase-associated lipocalin (NGAL), which may facilitate earlier detection of kidney injury before changes in traditional markers like creatinine become apparent. However, despite growing knowledge, there remains considerable heterogeneity in acute kidney injury prevention protocols across healthcare institutions.

The present investigation aims to synthesize current evidence on pathophysiology, risk stratification, and management of perioperative acute kidney injury. We examine risk prediction models and the role of novel biomarkers, evaluate pharmacologic and non-pharmacologic preventive strategies, and highlight the importance of bundle-based, patient-specific approaches. Therefore, this review aims to highlight the importance of hemodynamic optimization, nephrotoxin avoidance, and multidisciplinary collaboration. By identifying modifiable risk factors and using precision medicine tools, clinicians can improve renal outcomes and reduce the burden of perioperative acute kidney injury in vulnerable surgical populations.

For consistency, this review uses the Kidney Disease: Improving Global Outcomes (KDIGO) criteria for AKI, defined as an increase in serum creatinine by ≥0.3 mg/dL within 48 h, a 1.5-fold increase within 7 days, or urine output < 0.5 mL/kg/h for ≥6 h [4]. AKI is distinct from Acute Kidney Disease (AKD), which extends from 7 days to 3 months, and chronic kidney disease (CKD), which is defined by abnormalities in structure or function lasting ≥ 3 months. Table 1 summarizes these definitions and diagnostic time frames [4].

### 1.1. Pathophysiology and Risk Factors of Perioperative AKI

#### 1.1.1. Pathophysiological Mechanisms

Perioperative AKI stems from a complex interplay of hemodynamic disruption, inflammatory responses, and surgical stress. A central initiating factor is renal hypoperfusion, which frequently results from anesthesia-induced vasodilation, intraoperative hypotension, or intravascular volume depletion. Under normal conditions, the kidneys maintain glomerular filtration through autoregulatory mechanisms—afferent vasodilation mediated by prostaglandins and efferent constriction mediated by angiotensin II. However, when mean arterial pressure falls below this autoregulatory threshold, sympathetic activation causes afferent vasoconstriction, sharply reducing renal blood flow. This mismatch leads to tubular ischemia, ATP depletion, oxidative stress, and, ultimately, nephron injury [5].

This phenomenon may progress to ischemia–reperfusion injury during intraoperative events such as massive blood loss or resuscitation. The resulting generation of reactive oxygen species (ROS) and mitochondrial dysfunction leads to tubular apoptosis and necrosis. Additionally, surgical trauma and systemic inflammation activate cytokine cascades and endothelial dysfunction, further compromising renal microcirculation and perpetuating injury [5].

Certain surgeries carry unique pathophysiologic burdens. Cardiopulmonary bypass (CPB), for instance, exposes blood to non-endothelial surfaces, triggering systemic inflammation, hemolysis, and free hemoglobin release, which generates ROS and causes tubular damage. CPB also promotes low-flow states and activates the renin–angiotensin–aldosterone system (RAAS), further decreasing renal perfusion [5]. Vascular procedures involving aortic cross-clamping may induce prolonged ischemia followed by reperfusion injury. Similarly, pneumoperitoneum during laparoscopic surgery increases intra-abdominal pressure, compressing renal vessels and lowering perfusion, although typically without long-term AKI in most patients [6,7].

These diverse mechanisms converge on the same vulnerable structures: renal tubules, interstitial microvasculature, and mitochondria. The downstream result is energy imbalance, maladaptive signaling, and systemic inflammatory response that may trigger multi-organ dysfunction [5,8].

While these mechanisms are common to most perioperative settings, their relative contribution differs between surgical populations. In cardiac surgery, cardiopulmonary bypass introduces unique stressors including hemolysis, systemic inflammation, and ischemia–reperfusion injury, making cardiac-surgery-associated AKI (CSA-AKI) a distinct entity [5,9]. By contrast, in non-cardiac surgery, hypotension, nephrotoxin exposure, and sepsis predominate as drivers of perioperative AKI [5]. Distinguishing between these groups is clinically important, as preventive strategies may vary according to the underlying pathophysiology. Reflecting this, many prevention trials and meta-analyses have focused on cardiac surgical patients, where AKI risk is highest, while evidence in broader perioperative populations remains comparatively limited.

#### 1.1.2. Patient and Procedure-Related Risk Factors

Multiple patient characteristics are associated with elevated perioperative AKI risk. These include advanced age, male sex (particularly in general surgery), African American race, hypertension, congestive heart failure, chronic kidney disease (CKD), diabetes mellitus, peripheral vascular disease, and obesity. Obesity may increase risk by promoting a pro-inflammatory state and endothelial dysfunction [5]. Preoperative serum creatinine above 1.2 mg/dL is associated with increased risk of postoperative AKI, particularly in abdominal and urologic procedures [7].

Procedural and intraoperative factors also contribute significantly. Emergency operations extended surgical duration, hypotension, blood loss, and exposure to nephrotoxic agents (e.g., NSAIDs, IV contrast) all increase AKI risk. CPB use, as noted, is particularly high-risk related to its multifactorial renal insults. Laparoscopic surgeries with transient intra-abdominal hypertension can reduce perfusion but are rarely associated with overt AKI, unless compounded by other factors [7].

To improve early detection, emerging biomarkers such as neutrophil gelatinase-associated lipocalin (NGAL), kidney injury molecule-1 (KIM-1), and the cell-cycle arrest markers TIMP-2 × IGFBP7 have shown promise. These markers may detect subclinical injury prior to serum creatinine elevation, offering a window for preemptive intervention. However, their sensitivity and specificity vary by context, and their clinical integration remains under investigation [10,11].

### 1.2. Strategies for Prevention of Perioperative AKI

#### 1.2.1. Preoperative Measures

Preventing perioperative acute kidney injury (AKI) begins with identifying and optimizing patient risks. Diagnosis of preexisting renal dysfunction is critical. Diagnosis of preexisting renal dysfunction is critical for effective prevention of perioperative AKI. Reliance on serum creatinine alone may be misleading in patients with low muscle mass, malnutrition, or chronic illness, where creatinine underestimates the degree of impairment [12]. In such cases, cystatin C provides a more accurate reflection of glomerular filtration and has been shown to improve AKI risk prediction compared to creatinine-based estimates [12,13]. In addition, screening for microalbuminuria and proteinuria offers further prognostic information and should be incorporated into preoperative evaluation whenever feasible [14,15].

Medication reconciliation is essential, and nephrotoxic agents such as NSAIDs, aminoglycosides, and loop diuretics should be withheld when clinically feasible to minimize renal stress [16]. The perioperative use of renin–angiotensin–aldosterone system inhibitors (RAASi) has been debated. However, a recent meta-analysis found that continuing RAASi into the perioperative period was associated with a lower incidence of postoperative AKI in adjusted analyses [17]. In patients identified with subclinical kidney disease, avoidance of nephrotoxic agents alone is insufficient; careful dose adjustment of renally eliminated drugs is also essential [18].

Chronic kidney disease remains one of the most important risk factors for perioperative AKI, warranting proactive fluid and hemodynamic strategies [19]. Risk stratification tools, including machine learning have demonstrated high predictive accuracy using these features alone [20].

Volume status should also be assessed and corrected before surgery. Hypovolemia may compound renal hypoperfusion during anesthesia induction or surgical manipulation, particularly in elderly or diuretic-exposed patients. Guidelines recommend confirming euvolemia preoperatively when possible [16].

Pharmacologic preconditioning has also shown potential benefit. A 2024 meta-analysis of 15 trials reported that perioperative intravenous amino acid infusion significantly reduced AKI incidence, especially in cardiac surgery patients, and improved postoperative renal function markers [21]. This may reflect improved perfusion, oxygenation, and recruitment of renal functional reserve under stress.

Finally, cardiac surgical patients are particularly vulnerable due to the bidirectional interaction between heart and kidney function, known as cardiorenal syndrome [22]. This interplay amplifies perioperative risk and emphasizes the need for tailored preventive strategies in this high-risk group.

#### 1.2.2. Intraoperative Measures

Hemodynamic management remains critical during surgery. Avoiding hypotension, particularly mean arterial pressure (MAP) below 65 mmHg, is associated with reduced AKI risk. Goal-directed fluid and perfusion strategies are increasingly recommended [23]. A meta-analysis of 86 randomized trials demonstrated that goal-directed perfusion targeting oxygen delivery during cardiopulmonary bypass reduced cardiac surgery–associated AKI by 45% [24].

Remote ischemic preconditioning (RIPc), despite small-study limitations, has shown a modest but statistically significant reduction in AKI incidence (RR 0.86) across 31 trials and remains a low-cost, non-invasive strategy with potential benefit [24].

In contrast, agents such as sodium bicarbonate, mannitol, and N-acetylcysteine have not consistently demonstrated benefit. Recent reviews and guideline updates do not recommend them for routine use in AKI prevention [16,24,25]. KDIGO and other expert reviews emphasize minimizing exposure to intraoperative nephrotoxins, including certain contrast agents and synthetic colloids such as hydroxyethyl starch, related to their known renal risks [16,26]. Balanced crystalloids are preferred, with careful monitoring of fluid status and cardiac output.

Several network meta-analyses in cardiac surgery populations have compared pharmacologic and non-pharmacologic preventive interventions. In the largest to date, Chen and colleagues analyzed 161 randomized controlled trials involving 46,619 patients and identified nine effective strategies. Natriuretic peptides emerged as the most effective pharmacologic option (OR ≈ 0.30), while remote ischemic preconditioning (RIPC) was the only effective non-pharmacologic intervention (OR ≈ 0.76), both significantly reducing the incidence of post-cardiac surgery AKI, ICU length of stay, dialysis-dependent AKI, and mortality [27]. These findings complement evidence from bundle-based approaches such as KDIGO and ERACS, suggesting that while multimodal bundles provide broad preventive benefit, select single interventions like natriuretic peptides and RIPC may also contribute to improved perioperative renal outcomes.

#### 1.2.3. Postoperative Measures

Early recognition and intervention are essential. Monitoring serum creatinine and urine output, especially within the first 48 h, is vital. In biomarker-guided trials, early initiation of a KDIGO care bundle, typically including fluid optimization, avoidance of nephrotoxins, and hemodynamic monitoring, in patients with elevated urinary TIMP-2·IGFBP7 (a cell-cycle arrest biomarker indicating tubular stress) led to improved urine output and smaller creatinine rises [28,29].

Although biomarkers such as neutrophil gelatinase-associated lipocalin (NGAL), kidney injury molecule-1 (KIM-1), and the cell-cycle arrest marker panel TIMP-2×IGFBP7 can detect subclinical AKI earlier than creatinine, their clinical utility remains limited. High assay costs, logistical constraints surrounding point-of-care implementation, and a lack of standardized testing platforms currently restrict their deployment primarily to research environments or high-risk surgical cohorts [30].

Avoiding postoperative insults such as over-diuresis or nephrotoxin exposure is also important. A pilot trial found that empagliflozin started preoperatively reduced AKI risk in cardiac surgery without increasing adverse events like ketoacidosis [31].

Current guidance supports using integrated bundles, fluid optimization, glycemic control, and nephrotoxin avoidance, as the most effective strategy to reduce perioperative AKI [16,25,28,29].

Recent trials have demonstrated that combining early biomarker-based risk stratification, such as TIMP-2×IGFBP7, with structured KDIGO care bundles reduces AKI severity and has been associated with lower rates of MAKE-30 outcomes, dialysis, and mortality in high-risk surgical patients [28,29]. In a multicenter trial of high-risk cardiac surgery patients, implementation of a KDIGO bundle significantly reduced the incidence of moderate-to-severe AKI, emphasizing the feasibility and benefit of this approach in perioperative care [32]. However, a recent meta-analysis notes that most bundle trials have focused on general or mixed populations, with fewer studies addressing cardiac surgery and ICU patients despite their disproportionately high AKI risk [33].

### 1.3. Clinical Management of Established AKI

#### 1.3.1. Diagnostic Workup

Early recognition of acute kidney injury (AKI) relies on close monitoring for reduced urine output and rising serum creatinine. Once AKI is suspected, clinicians must evaluate for prerenal, intrinsic, and postrenal causes. Prerenal AKI, often due to hypovolemia or hypotension, generally responds to fluid administration. Intrinsic AKI may involve structural injury such as acute tubular necrosis, suggested by abnormal findings on urine microscopy. Postrenal AKI should be excluded using imaging, including bladder scan or renal ultrasound [5].

Novel biomarkers may assist in early differentiation, particularly in the perioperative setting where traditional markers lag behind injury onset. Urinary TIMP-2·IGFBP7, a pair of cell-cycle arrest markers expressed in response to tubular stress, has shown predictive value in detecting subclinical AKI before creatinine rises, enabling earlier intervention [28,29]. Additional markers like NGAL and KIM-1 continue to show promise but have not yet been widely adopted in clinical practice [10,11].

Implementation of automated AKI e-alert systems has been studied as a strategy to prompt earlier recognition and standardized responses. Meta-analyses show they reliably increase AKI documentation and dialysis use, reflecting earlier recognition and intervention, but these changes have not consistently translated into improved survival or renal recovery [34]. Their effectiveness likely depends on integration with structured care bundles, highlighting the need for better coupling of alerts with actionable management protocols.

#### 1.3.2. Supportive Management

Supportive care remains the cornerstone of managing established AKI. Fluid balance must be carefully individualized to avoid both hypoperfusion and volume overload. In adequately resuscitated patients, excessive fluid accumulation has been associated with increased morbidity and delayed recovery [35,36]. The REVERSE-AKI pilot trial demonstrated that a restrictive fluid management (RFM) strategy, targeting negative fluid balance post-AKI diagnosis, reduced the need for renal replacement therapy (RRT) and lowered adverse event rates without compromising hemodynamic stability [36]. This is consistent with a 2023 scoping review, which found that positive fluid balance is consistently linked to worse renal outcomes, though no consensus exists on optimal protocols [35].

Electrolyte and acid-base disturbances are frequent and require prompt correction. Hyperkalemia, metabolic acidosis, and volume status abnormalities should be addressed in parallel, with sodium bicarbonate typically reserved for severe acidosis (pH < 7.2) in patients who are not volume-overloaded [16].

Equally important is the avoidance of additional nephrotoxic insults. Potentially harmful agents, such as NSAIDs, aminoglycosides, and iodinated contrast, should be discontinued or avoided when possible. Implementation of KDIGO bundle principles, which emphasize nephrotoxin avoidance, hemodynamic optimization, and close monitoring, has shown promise in biomarker-guided trials [28,29].

#### 1.3.3. Renal Replacement Therapy

Renal replacement therapy is indicated in perioperative AKI when complications such as refractory fluid overload, hyperkalemia, severe acidosis, uremic symptoms, or persistent oliguria arise despite conservative management [16]. While timely initiation is critical in unstable patients, early RRT in the absence of clear indications has not demonstrated survival benefit. The STARRT-AKI trial, a multicenter randomized study of over 3000 critically ill patients, found that accelerated RRT initiation did not reduce 90-day mortality or improve recovery compared to a standard, delayed approach [37]. These findings support current guideline recommendations to tailor RRT timing based on clinical context rather than fixed thresholds [16].

Continuous renal replacement therapy (CRRT) is generally preferred for hemodynamically unstable postoperative patients due to its superior hemodynamic tolerance, whereas intermittent hemodialysis may be appropriate for stable patients [16]. In volume-overloaded individuals, restrictive fluid strategies like those in the REVERSE-AKI trial may reduce the need for RRT altogether [36].

Two contemporary meta-analyses and one IPD meta-analysis converge on the same conclusion. A 13-trial RCT meta-analysis (n = 5193) found no reduction in 28-day or overall mortality and no increase in renal recovery with early RRT, but higher hypotension (RR 1.34, 95% CI 1.17–1.53) and more infections (RR 1.83, 95% CI 1.11–3.02) [38]. A trial-sequential analysis RCT meta-analysis similarly showed no survival benefit and more RRT-related adverse events with early initiation [39]. Complementing these, an individual-patient-data meta-analysis of randomized trials showed no 28-day mortality difference (RR 1.01, 95% CI 0.91–1.13) and that ~42% of patients assigned to delayed strategy never required RRT, underscoring the value of watchful waiting when urgent indications are absent [40]. This data, together with STARRT-AKI, support an individualized, indication-driven approach and caution against routine “very early” initiation, while also avoiding excessive postponement (per AKIKI-2).

The timing of renal replacement therapy initiation in perioperative and critically ill patients with AKI has been extensively studied yet remains controversial. The ELAIN trial [41], a single-center RCT, reported that early initiation of RRT at KDIGO stage 2 with elevated NGAL significantly reduced 90-day mortality and improved renal recovery compared to a delayed approach. In contrast, the AKIKI trial [42], a large multicenter study, found no survival benefit with early initiation at KDIGO stage 3 compared to a delayed strategy, and nearly half of patients in the delayed arm never required RRT, thereby avoiding unnecessary exposure. More recently, the AKIKI-2 trial [40], tested two delayed strategies and showed that excessive postponement of RRT, waiting beyond 72 h of oliguria or until very high urea thresholds, resulted in no additional benefit and was associated with higher mortality. These pivotal trials highlight that while very early initiation may benefit selected high-risk patients, routine early initiation for all is not supported, and overly delayed initiation may be harmful. Current evidence supports an individualized approach guided by the patient’s clinical trajectory and the presence of urgent indications.

### 1.4. Patient Outcomes

#### 1.4.1. Short-Term Outcomes

Perioperative AKI is strongly associated with worse short-term outcomes, including prolonged hospitalization, greater resource use, and higher early mortality. In a retrospective cohort of patients undergoing major abdominal surgery, the presence of postoperative AKI was associated with a 12.6-fold increased risk of in-hospital death, along with significantly longer ICU and hospital stays [1]. The clinical burden of AKI is further underscored by its contribution to major adverse kidney events within 30 days (MAKE-30), including the need for dialysis and persistent renal dysfunction. See et al. demonstrated that implementation of KDIGO care bundles led to a significant reduction in MAKE-30 incidence, highlighting the value of structured early intervention in perioperative settings [43]. Additionally, Schaubroeck et al., in a recent meta-analysis, reported that care bundles reduced AKI severity and ICU stay across multiple trials [44]. Importantly, AKI duration, not just occurrence, has been identified as an independent predictor of short-term mortality, emphasizing the importance of early reversal and renal recovery [45].

#### 1.4.2. Long-Term Implications

Although frequently viewed as an acute and reversible complication, perioperative AKI has substantial long-term consequences. Persistent or prolonged AKI increases the risk of progression to chronic kidney disease (CKD), cardiovascular morbidity, and late mortality [16,46]. Mehta et al. found that patients with longer durations of AKI had a significantly increased risk of developing CKD, congestive heart failure, and myocardial infarction, with a pooled relative risk of 2.28 for long-term mortality compared to those without AKI [45]. Similarly, Ye et al. reported that patients who received nephrology follow-up after an AKI episode had significantly lower rates of CKD progression and long-term mortality than those who did not receive follow-up care [47].

These findings reinforce the importance of structured outpatient nephrology transition, particularly in high-risk surgical populations. Among elderly patients, especially those treated with continuous renal replacement therapy (CRRT), long-term outcomes are especially poor. Rhee et al. found that 6-month mortality exceeded 60% in patients over age 75 who received CRRT for AKI, suggesting the need for age-stratified goals of care discussions postoperatively [48].

Structured post-AKI and post-AKD care pathways, including nephrology follow-up, medication review, and CKD surveillance, are increasingly recognized as critical for improving long-term outcomes. Recent work on electronic alert systems, such as the AKIDS platform, demonstrates that automated outpatient detection of AKD can identify high-risk patients months before CKD progression or kidney failure, yet fewer than 3% are referred for nephrology follow-up, underscoring a substantial gap in care and opportunity for system-level intervention [30].

Despite these risks, referral for nephrology follow-up after AKI or AKD remains underutilized. A large VA cohort found that fewer than one in five AKI survivors with persistent kidney dysfunction were referred for outpatient nephrology care [49]. Similarly, a systematic review and meta-analysis of nearly 9000 patients reported a 22% reduction in long-term mortality with post-AKI nephrology follow-up, though effects on renal outcomes were heterogeneous and the evidence remains observational [47]. Together, these findings highlight both the survival benefits of specialized follow-up and the urgent need to improve referral patterns.

Between AKI and CKD lies an intermediate phase termed AKD, defined as persistent kidney dysfunction lasting more than 7 days but less than 3 months [4]. After cardiac surgery or critical illness, many patients progress into this stage, which strongly predicts later CKD and cardiovascular events. Recognition of AKD emphasizes the importance of structured post-discharge monitoring and nephrology follow-up to interrupt progression.

Renin–angiotensin system inhibitors are critical in managing conditions like heart failure, diabetes, and CKD, yet these are often withheld during acute kidney injury. Emerging observational evidence now supports a proactive approach to reinitiating RAASi after AKI recovery. In a target-trial emulation of CKD patients, Hattori et al. found that restarting RASi within a year was associated with a 15% lower risk of composite kidney outcomes and a 30% reduction in all-cause mortality, without increased hyperkalemia [50]. Conversely, Janse et al. reported that patients who discontinued RASi post-AKI had an elevated risk of death, myocardial infarction, or stroke (HR 1.13), while recurrent AKI rates were similar [51]. In large cohort studies from England and Sweden, Bidulka et al. found that continuing ACEI/ARB after AKI was not linked to higher risks of heart failure or recurrent AKI, although stopping was associated with increased mortality risk in England [52].

This data suggests that, once renal function stabilizes and risks are manageable, resuming RAAS inhibition may confer cardiovascular and renal benefits. While robust RCT data are lacking, these observational findings reinforce a targeted, patient-centered approach to ACEI/ARB resumption in post-AKI care.

#### 1.4.3. Patient-Reported Outcomes

Despite growing recognition of AKI’s clinical burden, patient-reported outcomes remain underexplored. Recent qualitative and survey-based research has revealed substantial emotional, physical, and informational impacts among AKI survivors. In a national survey, 84% of respondents reported their AKI episode had a highly negative impact on physical or emotional health, while only 52% rated their care team’s communication as “very good” [53]. Participants cited significant concerns about work and family, and many expressed confusion about their diagnosis and prognosis. Diamantidis et al. echoed these findings, identifying themes of diagnostic unawareness, fragmented post-discharge care, and a lack of education around recovery and self-management [54]. These findings suggest that current perioperative care pathways often fail to adequately address patient-centered experiences following AKI, highlighting an urgent need for improved education, transition planning, and patient engagement. Table 2 summarizes representative studies that have examined the clinical and patient-reported outcomes associated with perioperative AKI. Together, these findings illustrate the multidimensional burden of AKI on both survival and quality of life.

### 1.5. Comparing Effectiveness of Prevention Strategies

Fluid choice plays a pivotal role in mitigating the risk of perioperative AKI. Among crystalloids, balanced solutions like lactated Ringer’s and Plasma-Lyte have shown advantages over normal saline due to their lower chloride content, which helps prevent hyperchloremic metabolic acidosis and associated renal vasoconstriction. Studies comparing normal saline and balanced crystalloids in surgical populations reveal reduced rates of AKI and renal replacement therapy in those receiving balanced fluids [55].

Colloid use, particularly hydroxyethyl starch, has been controversial due to evidence linking it with increased AKI and higher mortality in critically ill and surgical patients [56]. Albumin, though physiologically favorable in volume expansion and oncotic pressure, remains limited by cost and mixed clinical outcomes. While some evidence suggests albumin may improve outcomes in septic patients, its benefit in perioperative AKI prevention remains inconclusive [57].

Maintaining renal perfusion through appropriate vasopressor support is critical during intraoperative hypotension. Norepinephrine remains the vasopressor of choice due to its balanced alpha and modest beta agonism, effectively supporting mean arterial pressure without excessive vasoconstriction [58]. Comparisons between norepinephrine and vasopressin reveal no significant differences in renal outcomes, though vasopressin may be preferable in vasodilatory shock states or in patients with right heart dysfunction [59]. Phenylephrine, a pure alpha-agonist, may compromise renal perfusion by increasing afterload and is typically avoided when renal protection is a priority [60].

Pharmacologic strategies to prevent AKI have yielded mixed results. Statins have demonstrated renoprotective effects in cardiac surgery patients, potentially through anti-inflammatory and endothelial-stabilizing mechanisms, yet findings remain inconsistent and context-dependent [61]. Dopamine, once widely used for renal protection, has shown no benefit and potential harm in multiple trials, leading to recommendations against its use [62]. Similarly, fenoldopam, a selective dopamine-1 receptor agonist, initially showed promise but failed to consistently demonstrate improved renal outcomes in large, randomized trials [63]. Diuretics like furosemide do not prevent AKI and may worsen outcomes if used in volume-depleted patients [64].

Emerging technologies for renal monitoring offer new avenues for AKI prevention. Near-infrared spectroscopy allows continuous, non-invasive measurement of renal tissue oxygenation, providing early indicators of perfusion deficits [65]. Intraoperative implementation of such tools may allow real-time interventions to restore perfusion and mitigate ischemic injury. Additionally, personalized hemodynamic monitoring using tools such as pulse pressure variation or dynamic arterial elastance enables titration of fluids and vasopressors based on individual physiologic responses rather than static parameters [66]. These technologies support precision perioperative management and may reduce renal insult in vulnerable patients.

The comparative effectiveness of prevention strategies for perioperative AKI underscores the need for a multimodal approach tailored to patient-specific risk factors. Balanced crystalloids are favored over saline, vasopressors must be carefully selected, and drug-based strategies require nuanced application of current evidence. Novel monitoring technologies offer promising adjuncts for early detection and individualized care. Ongoing research should aim to refine these tools and validate integrated protocols that encompass these various interventions for optimal renal protection. Table 3 summarizes notable clinical trials evaluating pharmacologic and non-pharmacologic strategies aimed at reducing the incidence and severity of perioperative acute kidney injury. These studies span diverse surgical populations and critical care contexts.

## 2. Discussion

Peri-operative AKI remains a common yet potentially preventable contributor to morbidity, length of stay, and progression to chronic kidney disease. The evidence reviewed here underscores three recurring themes: early identification of high-risk patients, meticulous hemodynamic and fluid stewardship, and rapid interdisciplinary response when sub-clinical renal stress is detected.

Single interventions rarely provide durable protection; instead, success comes from coordinated “renal-protective bundles” that blend balanced crystalloid resuscitation, norepinephrine-guided vasopressor support, strict avoidance of nephrotoxins, and early nephrology input [55,57]. Adherence to such bundles has cut AKI incidence by 20–30% in mixed surgical cohorts [68].

Traditional static markers: central venous pressure or urine output, often lag actual renal hypoperfusion. Dynamic indices (stroke-volume variation, pulse-pressure variation) and tissue-level technologies such as near-infrared spectroscopy detect malperfusion hours earlier [66]. When clinicians intervene based on these signals, e.g., small fluid bolus or vasopressor titration creatinine rise and RRT rates fall [69].

Large RCTs have debunked low-dose dopamine and most statin “renoprotective” hypotheses, even hinting at potential harm in high-dose peri-operative statin regimens [62,70]. Fenoldopam shows promise only in highly selected, contrast-exposed cardiac patients and requires blood-pressure vigilance [63]. Consequently, pharmacotherapy should be individualized and never substitute for sound fluid and hemodynamic practice.

Heterogeneous AKI definitions and short follow-up windows cloud many legacy studies [68]. Next-generation trials must (i) use KDIGO-based, biomarker-augmented endpoints; (ii) stratify by surgical type and pre-existing CKD; and (iii) capture 90-day renal recovery and quality-of-life data. Machine-learning risk models already outperforming clinical scores in ICU cohorts [71] should be prospectively validated to trigger bundle deployment automatically.

Balanced crystalloids for volume, norepinephrine for pressure, avoidance of nephrotoxins, and real-time organ-specific monitoring form today’s best-supported AKI-prevention toolkit. With rigorous implementation and ongoing investigative refinement, meaningful reductions in peri-operative AKI are within reach.

Despite progress, several limitations remain. Heterogeneity in AKI definitions, inclusing KDIGO, RIFLE, or institution-specific thresholds, complicates comparison across studies. Many trials are small, single-center, or limited to short-term endpoints, making it difficult to draw conclusions about long-term renal trajectories. Biomarkers such as NGAL, KIM-1, and TIMP-2×IGFBP7 are promising but remain limited by cost, availability, and lack of standardization across institutions. Cystatin C may provide a more reliable alternative to creatinine in selected perioperative patients, but its role in prediction algorithms requires further validation. Similarly, perioperative proteinuria screening is underutilized, despite consistent associations with postoperative AKI risk. In response to recent trials and systematic reviews, we incorporated discussion of network meta-analyses in cardiac surgery, biomarker-guided bundle approaches, electronic alert systems, and structured post-AKI/AKD care. Together, these additions highlight not only traditional prevention strategies but also emerging system-level interventions that address recognition, risk stratification, and long-term follow-up.

Future research should prioritize multicenter, biomarker-guided randomized trials with standardized KDIGO endpoints and long-term follow-up. Precision-medicine tools, including machine-learning prediction models that integrate cystatin C, proteinuria, and dynamic hemodynamic variables, may enable automatic risk stratification and timely bundle deployment. Cardiorenal interactions in cardiac surgery deserve focused investigation, given the unique interplay between low cardiac output, venous congestion, and renal perfusion. Finally, patient-centered outcomes such as quality of life, education, and structured follow-up remain understudied, despite their importance for recovery and long-term prognosis.

## 3. Conclusions

Perioperative acute kidney injury remains a frequent and clinically significant complication that worsens both short- and long-term surgical outcomes. Current evidence supports an integrated prevention strategy focused on rigorous preoperative risk stratification, balanced crystalloid resuscitation, individualized hemodynamic targets, and strict nephrotoxin avoidance. These bundle-based approaches, when implemented systematically, have consistently reduced AKI incidence across diverse surgical populations.

Looking forward, advances in biomarker panels, real-time perfusion monitoring, and machine-learning risk prediction hold promise for earlier detection and more precise intervention. Multidisciplinary pathways that actively involve anesthesiologists, surgeons, and nephrologists will be essential for translating these innovations into routine practice. By combining evidence-based prevention bundles with emerging precision-medicine tools, clinicians can meaningfully reduce perioperative AKI and improve long-term renal and cardiovascular outcomes.

## Figures and Tables

**Table 1:** KDIGO definitions and diagnostic time frames for AKI, AKD, and CKD.

Condition	Definition/Criteria	Time Frame
AKI	Increase in serum creatinine by ≥0.3 mg/dL within 48 h or ≥1.5× baseline within 7 days or urine output < 0.5 mL/kg/h for ≥6 h [4]	≤7 days
AKD	Persistence of AKI criteria or eGFR < 60 mL/min without meeting CKD criteria [4]	>7 days to <3 months
CKD	Structural or functional kidney abnormality (e.g., proteinuria, imaging, biopsy findings) or eGFR < 60 mL/min for ≥3 months [4]	≥3 months

**Table 2:** Clinical Outcomes Following Perioperative Acute Kidney Injury.

Study	Population/Exposure	Key Findings	Clinical Implications
O’Connor et al. [1]	Patients undergoing major abdominal surgery with postoperative AKI	AKI associated with a 12.6× increase in mortality, longer ICU and hospital stays, and higher complication rates	Perioperative AKI is a critical determinant of early adverse outcomes and resource utilization
Mehta et al. [45]	Meta-analysis of 18 studies assessing AKI duration	Longer AKI duration significantly associated with increased long-term mortality (RR 2.28), CHF, MI, and CKD progression	AKI duration should be considered in prognostic models and discharge planning
See et al. [43]	Critically ill patients receiving KDIGO care bundles, with or without biomarkers	Bundle use led to significant reductions in MAKE-30, RRT need, and short-term mortality	Standardized bundle implementation can mitigate short-term AKI complications
Ye et al. [47]	AKI survivors with vs. without post-discharge nephrology follow-up	Follow-up associated with lower long-term mortality and reduced CKD progression	Structured nephrology care improves post-AKI outcomes and supports transition planning
Switzer et al. [53]	Online survey of 124 AKI survivors via AAKP	84% reported severe physical and emotional impact, 67% had family/work concerns, only 52% rated provider communication as “very good”	Highlights gaps in education, discharge counseling, and patient engagement post-AKI

**Table 3:** Selected Interventions to Prevent or Mitigate Perioperative Acute Kidney Injury.

Author (Year)	Groups Studied and Intervention	Results and Findings	Conclusions
Zarbock (2021) [67]	Cardiac surgery patients; Remote ischemic preconditioning vs. control	RIPC group had significantly lower incidence of AKI; fewer needed dialysis	Supports RIPC use in high-risk cardiac patients
Semler (2022) [55]	ICU patients; Balanced crystalloids vs. saline	Balanced fluids reduced incidence of AKI and need for renal replacement therapy	Balanced crystalloids preferred over saline in critical care
Argalious (2023) [61]	Vascular surgery patients; Preoperative statins vs. no statins	Lower rate of postoperative AKI in statin group	Preoperative statins may reduce AKI risk
Russell (2023) [59]	Patients with vasodilatory shock; Vasopressin vs. norepinephrine	Both agents maintained MAP, vasopressin group had lower creatinine rise	Vasopressin may offer renal benefits in select patients
